# Persistent consumer response to a nationwide food safety recall in urban India

**DOI:** 10.1093/qopen/qoac025

**Published:** 2022-09-20

**Authors:** Cherry Law, Laura Cornelsen

**Affiliations:** Department of Agri-Food Economics and Marketing, School of Agriculture, Policy and Development, University of Reading, Whiteknights, RG6 6EU, Reading, UK; Population Health Innovation Lab, Department of Public Health, Environments and Society, Faculty of Public Health and Policy, London School of Hygiene & Tropical Medicine, London WC1E 7HT, UK; Population Health Innovation Lab, Department of Public Health, Environments and Society, Faculty of Public Health and Policy, London School of Hygiene & Tropical Medicine, London WC1E 7HT, UK

**Keywords:** Food safety, Instant noodles, Recall, Event-study, India

## Abstract

Little is known about consumer response to food safety recalls in low- and middle- income countries. Using an event-study framework, this paper examines the immediate and long-term changes in noodle purchases after the nationwide removal of Maggi instant noodles from the market in India in 2015. We show that this recall had a negative impact on the purchases of Maggi noodles among urban households for at least two years. This provides evidence of the huge costs of recalls on food producers that can be leveraged by policymakers to promote food safety. We also find strong evidence for a positive spillover effect to non-Maggi noodles that is more persistent among households with more regular purchasing habits of Maggi noodles. This indicates that consumers with more persistent habits of buying a recalled product are less likely to stigmatize alike food products under different brands. Our results are robust to alternative assumptions of pre-trends in purchases and placebo tests.

## 1 Introduction

With rising income and improved access to media, there has been an increasing pressure for policymakers to improve the safety of food available in low- and middle- income countries (LMICs) (Jaffee et al. 2018; Hoffmann et al. 2019). Nonetheless, they often face resource and capacity constraints that may limit their ability to perform inspections and tests, and to monitor the compliance of food producers effectively.

Economic literature has argued that the potential costs and reputation effects from food recalls can incentivize firms to exert efforts to ensure food safety, reducing the need to ensure compliance through frequent inspection and regulation enforcement (Thomsen & McKenzie 2001; Starbird 2005; Taylor et al. 2016; Hoffmann et al. 2019). The negative effects of food recalls on the demand for affected products are well documented in high-income settings (e.g. Marsh et al. 2004; Arnade et al. 2009). There is also evidence on lost firm profitability as reflected by the reduction in stock market value of implicated firms after recalls (Salin & Hooker 2001; Pozo & Schroeder 2016; Ollinger & Houser 2020). Indeed, firms may face substantial profitability losses if the recalls have a persistent reputation effect and hence reduce the long-term demand for their products (Pozo and Schroeder 2016). Costly marketing campaigns may be needed to recover their reputation (Byun et al. 2020), further incentivizing firms to prevent the occurrence of safety recalls. However, it is unclear to what extent policymakers in LMICs can leverage these reputation effects to promote food safety as little is known about how demand patterns in these settings may evolve over time in response to recalls.

To understand the implications of food safety recalls in LMICs, this paper examines the immediate and long-term consumer response to a food safety scandal in India. In June 2015, Maggi noodles^1^ were recalled and banned temporarily across India due to the controversy over its excessive level of lead and presence of monosodium glutamate (MSG) (Financial Times 2015). This (the Scandal here after) was widely covered by news media, leading to a significant public outcry (BBC 2015; Business Insider India 2015; The Washington Post 2015). The sales of Maggi noodles plummeted by 90 per cent within a month (The Times of India 2015a). About 400 million packets of Maggi noodles were reportedly destroyed (Nestlé 2016a). These products were only reintroduced into the market in November 2015 when they passed a new round of laboratory tests. The widespread public dissatisfaction and the sale ban of Maggi noodles are a clear example of the rising awareness of food safety in LMICs.

The present paper draws upon a household-level panel dataset of packaged noodle purchases in urban India between 2013 and 2017. Leveraging on the exogeneity of the Scandal, we compare the changes in packaged noodle purchases by households who bought Maggi noodles (buyer) regularly prior to the Scandal with households who never bought Maggi noodles (comparison). Since this construction of comparison group prevents direct identification of the Scandal impacts on Maggi noodles, we estimate the disproportionate effects of the Scandal on monthly purchases of all packaged noodles and non-Maggi packaged noodles by the buyer households. From the findings on these two outcomes, we are able to infer changes in Maggi noodles purchases among the buyer.

We focus on studying purchase changes among regular customers of Maggi noodles as brand commitment can make them more resilient to negative information and thus reduce the magnitude of profitability losses for food firms (Ahluwalia et al. 2000; Dawar & Pillutla 2000; Pullig et al. 2006; Byun et al. 2020 ). Evidence on heterogeneous consumer response by purchasing habits would therefore improve our understanding of the implications of recalls and help policymakers to design effective food safety regulations. This paper identifies regular customers as households who purchased Maggi noodles once every month in the 24 months prior to the Scandal (i.e. monthly buyer). We further classify remaining households into two groups, based on the number of months they purchased Maggi noodles between May 2013 and April 2015: frequent buyer (13–23 months) and infrequent buyer (1–12 months). An event study approach is then used to examine the immediate and long-term changes in noodle purchases among these buyer groups in response to the Scandal. To deal with the potential selection from the assignment of buyer status, we apply household fixed effects to account for household heterogeneity and inverse propensity weighting to enhance the comparability between the buyer and comparison groups.

Our results show a dramatic decrease in all noodle purchases of all buyer groups relative to the comparison group during the Scandal period while the non-Maggi noodle purchases by monthly and frequent buyers increased significantly. These consumer response provide evidence in support of a substantial negative effect of the Scandal on purchases of Maggi noodles. The decline in all packaged noodle purchases remained statistically significant over the two years after Maggi noodles returned to the market, particularly among monthly buyer. Combined with the absence of long-term reductions in non-Maggi noodles purchases, this suggests that the Scandal led to a persistent decrease in Maggi noodle purchases among all households who had bought these products previously. These results are possibly an underestimate if the Scandal also triggered concerns over non-Maggi noodles among comparison households who subsequently reduced purchases of these products. Nevertheless, our findings are robust to alternative assumptions of the pre-Scandal purchase trends, and hence are unlikely to be sensitive to potential time-varying unobservables. Placebo tests confirm that there were no significant changes in purchases of other processed foods during and after the Scandal.

This paper contributes to two strands of literature. First, there is a paucity of empirical research regarding the consumer response to food safety recalls in LMICs. Findings from this paper provide evidence for negative demand effects of recalls in LMICs which can be leveraged by policymakers to promote food safety. The persistent shift from Maggi noodles to other brands among the monthly buyer demonstrate that food producers failing to meet food safety regulations can potentially face substantial sale losses from their regular customers. Second, even though the effects of food safety recalls on affected products are widely studied in developed countries, limited attention is devoted to their spillover effects to non-recalled food brands. This paper adds to this body of knowledge by illustrating a positive effect of the Scandal on non-Maggi noodle purchases among the monthly and frequent buyers but a negative effect among the infrequent buyer. Our results shed light on how consumers draw inferences from information about food safety. They also indicate that consumers with more regular purchasing habits of a branded product are less likely to stigmatize other brands and thus highlight the benefits of branding in the food industry.

The reminder of this paper is organized as follows. The next section reviews literature on consumer response to food recalls, followed by the details of the Maggi Noodle Scandal. The subsequent sections describe the data and the empirical strategy, and then present the results. In the last section, we discuss our findings and their policy implications.

## 2 Related literature

A large body of existing studies has looked at consumer response to meat product recalls in developed countries.^2^ For example, Marsh et al. (2004) employ national level aggregate disappearance data in the US and find that the significant effects of recalls on the demand for affected meat products subsided within three quarters. They also find that those events had positive effects on the demand for their substitutes, which were; however, offset by an overall decrease in meat demand. Similar substitution effects are also found by Tonsor et al. (2010) using the same dataset from 1982 to 2007. They show that beef recalls in the US had a negative effect on beef demand but a positive effect on poultry demand during in the three-quarter period following recalls. A more recent study by Shang and Tonsor (2017) uses monthly grocery-scanner data and shows that beef E. coli recalls significantly reduced the demand for recalled ground beef contemporaneously in most US regions.^3^

Studies on the demand effect of non-meat recalls are less common. Arnade et al. (2009) investigate consumer response to the recall of spinach products in the US due to the outbreak of E.coli. Using national weekly retail scanner data, they show that the recall led to purchases shifting from spinach products to bulk lettuce of all types, but resulted in no long-term change in the demand for leafy greens as a whole. On the other hand, Toledo and Villas-Boas (2019) find no evidence of substitution in their study on consumer response to recalls of eggs in California during the 2010 Salmonella outbreak. Using a scanner level dataset from a national grocery chain and a difference-in-differences approach, they find a temporary reduction in overall egg sales after the recalls, which remained significant for at least three months. Taken together findings from meat products, food safety recalls generally have immediate and adverse demand effects on the implicated products. However, evidence on their effects on the demand for related products is rather mixed.

As previously discussed, the dynamics of consumer response over time is crucial in understanding the implications of recalls on food producers and thus their incentive to ensure food safety. Despite the considerable research interests in food recalls, only a few studies have examined the persistence of the adverse demand effects. Arnade et al. (2009) show that the shifting in demand among leafy green vegetables persisted over a period of 68 weeks after the spinach E.coli recalls, whereas Shang and Tonsor (2017) find that most of beef E. coli recalls only impacted ground beef demand for a very short period after the event. These differences in the persistence of demand effects might be explained by heterogeneity in products.

With the focus of demand changes at national level, many of the above studies do not take into account that consumer sensitivity to food recalls may vary. Literature on consumer response to media coverage has shown that persistent purchasing habits can mitigate the demand effects of negative product information, such as the announcements of bovine spongiform encephalopathy (BSE) cases (Ding et al. 2013). Using detailed household-level retail scanner data, Rieger et al. (2016) find strong empirical evidence that short-term marginal adjustments in demand for meat products, triggered by media coverage of the 2011 German Dioxin scandal, were over-compensated by habit persistence. If purchasing habits play a similar role in consumer response to food recalls, firms with a regular consumer base may not face high reputation risks from recalls, making them less incentivized to ensure the safety of their products.

Accounting for differences in purchasing habits is particularly important when examining the demand effects of branded product recalls. Customers who regularly purchase from the same brands can be less sensitive to product recalls (Ahluwalia et al. 2000; Pullig et al. 2006; Byun et al. 2020). Consumer response to branded food product recalls has not been widely studied empirically. Using market-level retail data in the US, Thomsen et al. (2006) find evidence for short-term sales losses among the recalled brands of Frankfurters along with increases in sales of non-recalled brands. Similar results can also be seen in the recall of Peter Pan peanut butter in the US. Bakhtavoryan et al. (2014) use time series data on aggregated household purchases of peanut butter and find that the recall was associated with negative impacts for the implicated brand and positive spillover effects for the leading competitor brands. Nonetheless, none of the studies consider the role of purchasing habits in the dynamics of demand responses.

Despite the limitations, the above studies from high-income settings provide important background for our study. They demonstrate that food recalls typically lead to short-term reductions in the demand for affected products. Across recall events, there are variations in the persistence of such reductions as well as the direction of spillover effects to related products. The extent to which consumers would stigmatize all products related to the recalled products influences the magnitude of collective reputation, which would in turn incentivize the industry to implement voluntary actions aimed at enhancing food safety (e.g. adoption of minimum quality standards) (Winfree & McCluskey 2005; Adalja et al. 2022). On the other hand, a positive spillover effect to non-recalled brands indicates a higher risk of lost market share from recalls and highlights the benefits of brandings in the food industry (Thomsen et al. 2006).

Similar empirical evidence on food product recalls in the context of LMICs is imperative for policymakers to understand the implications of recalls on firms and thus to promote food safety effectively. To produce such evidence, objective sales or purchase data collected before and after the recall are often required, which are rarely available in LMICs (Hoffmann et al. 2019). One exception is Peng et al. (2015) who find negative effects of a scandal reported by We Media, concerning set-style yogurt and jelly, on monthly sales in 12 supermarkets in Beijing although those products were not recalled.^4^ This paper fills this literature gap by using household-level purchase data to identify both the immediate and long-term effects of a large-scale branded product (Maggi noodles) recall on consumer purchases in urban India. We illustrate how these effects differed across consumers with varying purchasing habits and provide evidence on the spillover effects to non-Maggi noodles. Our study allows us to draw insights on the potential reputation effects over safety recalls faced by food producers in LMICs.

## 3 Maggi noodles scandal

Maggi noodles were introduced to India in the early 1980s by Nestlé India. They are packaged noodles that have been seen as an iconic Indian snack and consumed by Indians across all ages and socio-economic segments (Baviskar 2018). Maggi noodles had a 70 per cent share of an over 500million USD instant noodles market in India, as reported by The Times of India (2015b). In 2015, they faced a major food safety scandal due to controversy on higher than permissible level of lead and the presence of MSG found by a food safety laboratory in Uttar Pradesh. While MSG was not banned in India, Maggi noodles' package stated that the product had ‘No Added MSG’, violating the food labeling regulations in India (The Hindu 2015a). The public concerns began to develop on 21^st^ May after national news reported these food safety violations and the food safety body in Uttar Pradesh asked Nestlé India to recall the batch collected for the laboratory tests (Business Standard 2015). Meanwhile, the hashtag #MaggiBan started trending on social media. On 29^th^ May, the Indian government ordered the Food Safety and Standards Authority of India (FSSAI) to investigate these safety concerns.

While Nestlé India maintained that the lead level in Maggi noodles was within the permitted level, they were found unsafe in the laboratory tests conducted in Delhi on 2^nd^ June. The Delhi government subsequently banned Maggi noodles for 15 days while other Indian states ordered further tests (Financial Express 2015)*.* Over the next few days, Maggi noodles were banned in Gujarat, Uttarakhand, Tamil Nadu, Telangana, and Jammu and Kashmir (The Times of India 2015c). On 5^th^ June, Nestlé India announced the withdrawal of Maggi noodles across India despite asserting the products were ‘safe’ (Nestlé 2016b). During the same time, the FSSAI ordered the recall and production ban of all nine Maggi noodle variants since samples were found to be ‘unsafe and hazardous’ for humans (Business Standard 2015). Consequently, Nestlé India filed a judicial review of the FSSAI ban (Business Standard 2015).^5^

On 13^th^ August, the Court ruled that Maggi noodles could return to the market if fresh tests on existing samples were found safe (Nestlé 2016b). Later that month, Nestlé India released a series of video advertisements with the hashtag #WeMissYouToo on social media (The Hindu 2015b). Maggi noodles were cleared from the mandated laboratories in October 2015. On 9^th^ November, Maggi noodles were available for sale again throughout India (Nestlé 2016b). Nestlé India had a relaunch campaign ‘Welcome Back Maggi’ with ads reassuring the safety of their products (Choudhary 2015; Tandon 2016). New variants of Maggi noodles were introduced in 2016 to regain its market share (Business Standard 2015).

This food safety crisis of Maggi noodles caused substantial public outcry across Indian states and widespread coverage by international news (CNBC 2015; Financial Times 2015; Forbes 2015). While Maggi had regained status as the leading noodle brand by April 2016, its market share in India only recovered to 50 per cent, considerably lower than the 70 per cent prior to the Scandal (The Times of India 2015b; Dhanesh & Sriramesh 2018).

## 4 Data

We use purchase data from an on-going demographically representative urban household panel, collected by the market insight company, ‘Kantar—Worldpanel Division, India’ from May 2013 to November 2017.^6^ In this panel, over 60,000 households are invited to participate based on their occupational socio-economic status, age of the person responsible for food purchases as well as the state of domicile. The primary shoppers of the participating households fill in paper diaries to record all purchases of processed foods taken home. Purchases made for consumption outside of home are excluded.^7^ The paper diaries collect data on the brand of products purchased and the date and volume of purchases but not on monetary expenditure or prices. To ensure that purchases are recorded correctly, interviewers regularly check the information in the paper diaries against packaging and wrappers that are collected by households in pre-provided containers. Around 5 per cent of the households drop out from the panel each year. New participants are invited regularly to ensure that the panel remains representative.

**Figure 1.**
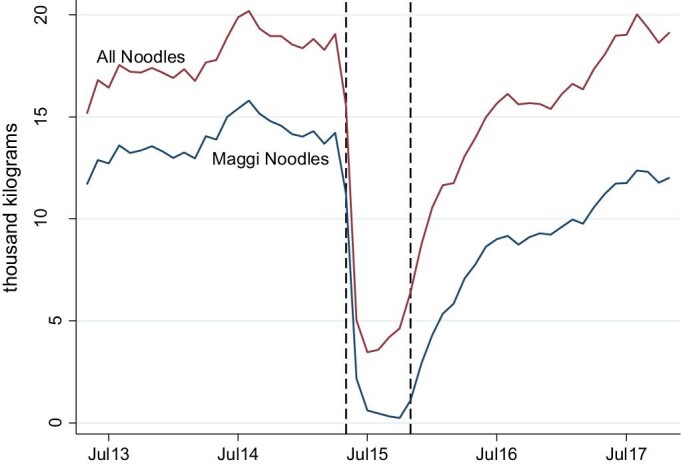
Total take home purchases of packaged noodles in urban India, May 2013–Nov 2017. Note: The dashed lines indicate the period from May 2015 to November 2015.

Fig. 1 represents the market-level take-home purchases of all packaged noodles and Maggi noodles, estimated with the purchase record specific weight given by the data company.^8^ The dashed lines indicate the Scandal period from May 2015 to November 2015, covering both the time when the food safety information was released and also when Maggi noodles were unavailable for sale. From May 2013 to April 2015, Maggi noodles represented over 75 per cent of packaged noodles bought by urban Indian households, with over 10,000 kilograms of Maggi noodles purchased every month. In May 2015, the monthly purchases of Maggi noodles decreased by tenfold and remained at a very low level (i.e. <1000 kg) over the next few months. The non-zero level of these purchases implies that some households still managed to buy Maggi noodles during the Scandal period despite the nationwide ban. This could be because some stores might not have taken them off the shelves immediately. It also took time to recall and destroy all the products. Nestlé India estimated that there were 27,420 tonnes of Maggi noodles in the factories, distribution centers, distributors, and market on 5^th^ June 2015, which would take at least 40 days to be destroyed (Nestlé 2016a). There is, however, no doubt that the availability of Maggi noodles was greatly reduced with Nestlé India reported to have destroyed over 400 million packets of its noodles in 2015 (Nestlé 2016a). The purchase of Maggi noodles started to recover in November 2015 when they were back on shelves, however, at a rate much slower than the drop in May 2015. The widening gap between the two solid lines from 2016 to 2017 indicates that relatively more non-Maggi noodles were bought than Maggi noodles. The share of Maggi noodles purchased was still down by more than 12 per cent points (around 62 per cent) by late 2017 compared to pre-Scandal period.

### 4.1 Purchase patterns of Maggi noodles

Next, we look at the purchases of Maggi noodles at household level. The data consists of 51,735 unique households who stayed in the panel for the whole period of interest. We remove observations from 1983 households who did not report their purchases consistently.^9^ An additional 3,372 households who never purchased any noodles are also dropped.

To explore difference in purchasing habits, households are classified into the following four groups, based on the number of months they had purchased Maggi noodles over the 24 months prior to the Scandal (i.e. May 2013 to April 2015)^10^:

- Monthly buyer: 24 months;
- Frequent buyer: 13–23 months;
- Infrequent buyer: 1–12 months and;
- Non-buyer: 0 months;

We consider the monthly buyer as the regular customers of Maggi noodles. This is similar to the approach used in Byun et al. (2020), who define ‘regular’ customers as those that made two or more purchases of the recalled products in the three months before the recalls occurred. Table 1 summaries the key demographic characteristics available in our dataset. It shows that the monthly and frequent buyers consist of relatively more households from higher socio-economic classes. On average, these two buyer groups have a larger household size compared to the infrequent and non-buyers. They also are more likely to have children above five-year-old.

**Table 1.** Descriptive statistics of household groups, 2013.

	Non-buyer	Infrequent buyer	Frequent buyer	Monthly buyer	All
Number of months purchased Maggi noodles in the 2 years prior to the Scandal	0	1–12	13–23	24	
Household size^1^	4.354	4.450	4.733	4.936	4.571
Socio -economic class^2,3^					
Upper class	0.211	0.298	0.470	0.605	0.373
Upper middle class	0.267	0.308	0.286	0.234	0.294
Middle class	0.325	0.274	0.183	0.112	0.235
Lower class	0.197	0.120	0.061	0.049	0.098
Presence of children in the following age groups in the household^3^					
Infant	0.023	0.025	0.029	0.033	0.055
1 year old	0.034	0.038	0.043	0.047	0.026
2–4 years old	0.100	0.120	0.152	0.152	0.133
5–9 years old	0.167	0.218	0.266	0.290	0.236
10–14 years old	0.235	0.291	0.349	0.403	0.314
15–17 years old	0.214	0.231	0.254	0.306	0.241
States^3^					
Delhi	0.021	0.023	0.059	0.225	0.042
Jharkhand	0.023	0.050	0.085	0.052	0.063
Andhra Pradesh	0.104	0.092	0.040	0.003	0.070
Maharashtra	0.090	0.118	0.126	0.109	0.120
Punjab/Haryana	0.007	0.027	0.149	0.218	0.080
West Bengal	0.124	0.067	0.064	0.050	0.068
Gujarat	0.080	0.069	0.044	0.023	0.058
Karnataka	0.067	0.074	0.033	0.009	0.056
Kerala	0.051	0.067	0.030	0.007	0.050
Rajasthan	0.031	0.037	0.028	0.023	0.033
Orissa	0.012	0.037	0.035	0.005	0.034
Madhya Pradesh	0.140	0.091	0.065	0.039	0.082
Uttar Pradesh	0.124	0.097	0.130	0.156	0.114
Tamil Nadu	0.100	0.114	0.057	0.012	0.088
Bihar	0.026	0.036	0.053	0.069	0.044
No of households	2450	23,862	18,618	1090	46,020

1 F-statistics of one-way ANOVA test is 96.38 (with *P*-value < 0.001),  indicating that the means are not equal across household groups.

2 The socio-economic class was classified by the data company based on the education level of the person responsible for food purchases and the number of durables owned by the household: *Upper class*—Minimum literacy/school up to 4 years & minimum 6 durables in their household; *Upper middle class* -Minimum literacy/school up to 4 years & minimum 5 durables in their household; *Middle class—*Minimum literacy/school up to 4 years & minimum 3 durables in their household; *Lower class—*Illiterate and no durable or minimum 1 durable in their home.

3 Chi^2^ test of distribution equality are rejected for socio-economic class, presence of children and states (*P*-value < 0.001).

**Figure 2.**
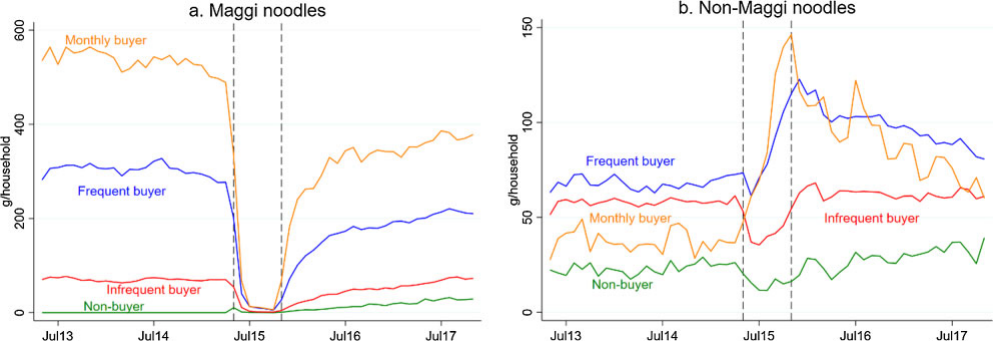
Average monthly purchases of packaged noodles across household groups, May 2013–November 2017. Note: The dashed lines indicate the Scandal period from May 2015 to November 2015. Household groups are classified based on their purchase frequency of Maggi noodles in the 24 months prior to the Scandal: 24 months for Monthly buyer; 13–23 months for Frequent buyer; 1–12 months for Infrequent buyer; 0 month for Non-buyer.

We present the differences in purchase level of Maggi and non-Maggi noodles across the household groups in Fig. 2a and 2b, respectively. Before the Scandal, apart from the monthly buyer, all household groups seemed to have an overall flat purchase trend of Maggi noodles (Fig. 2a). The monthly buyer purchased, on average, around 300g more Maggi noodles per month, compared to the frequent buyer. This difference shrunk to around 150g–200g after November 2015. Following a similar pattern as the estimated total purchases in Fig. 1, a sharp drop in monthly Maggi noodle purchases can be observed across all buyer groups during May–November 2015. The magnitude of this drop increased with the purchase frequency of Maggi noodles. Households purchasing Maggi noodles monthly prior to the Scandal displayed the largest decline in mid 2015 but the fastest recovery in purchases in late 2015. Interestingly, there was a slightly increasing trend in Maggi noodle purchases after the Scandal among the non-buyer though the volume purchased remained at the lowest among all groups. This could be due to the post-Scandal marketing campaigns run by Nestlé India.

Fig. 2b illustrates that the monthly buyer rapidly increased their purchases of non-Maggi packaged noodles during the Scandal and subsequently reduced these purchases gradually after November 2015. All other household groups showed an initial decrease in their monthly purchases of non-Maggi noodles in May 2015, which then increased in the following few months. Overall, the purchase pattern of non-buyer was more stable than other groups. These two descriptive figures suggest that there was a shift in purchases from Maggi to non-Maggi noodles and its magnitude was likely to vary depending on how often households bought Maggi noodles prior to the Scandal.

## 5 Empirical strategy

Our aim is to investigate how regular customers of Maggi noodles adjusted their noodle purchases over time in response to the Scandal. To do this, we use an event study approach and explore the consumer response across households with heterogeneous purchasing habits of Maggi noodles. As the Scandal triggered a ban of Maggi noodles across all states, the key empirical challenge in identifying consumer reactions is to isolate the effect of confounding events on noodle purchases. We deal with this issue by comparing the noodle purchases of those households that bought Maggi noodles with ones that did not. In this regard, we set the non-buyer households as the comparison group for each of the other three buyer groups. Since the non-buyer did not purchase any Maggi noodles at all for the two years prior to the Scandal, they would have little incentive to change their purchase patterns in response to the safety concerns over Maggi noodles. However, this set up could give rise to an identification concern as the non-buyer group is a selected subsample of the population who could be different from the buyer groups. Any differences in noodle purchases could reflect the selection bias rather than consumer response to the Scandal. In the following, we first introduce the event study specification and then discuss how we deal with the selection bias in more detail.

### 5.1 Event study specification

We follow an event-study difference-in-differences design, which identifies the effect of an intervention or event by eliminating the time-invariant differences between the treated (i.e. the buyer groups in our case) and comparison groups that can result from unobservables as well as the effects of time-variant unrelated events affecting both groups. Specifically, for each buyer group, we examine their immediate and long-term responses to the Scandal with the following specification:

(1) \begin{equation*} {\rm{\ }}{Y}_{th} = \ \alpha + \mathop \sum \limits_{\begin{array}{@{}*{1}{c}@{}} \scriptstyle{t = - 23}\\ \scriptstyle{t \ne - 1} \end{array}}^{30} {\tau }_t{D}_t + \gamma Buye{r}_h + \mathop \sum \limits_{\begin{array}{@{}*{1}{c}@{}} \scriptstyle{t = - 23}\\ \scriptstyle{t \ne - 1} \end{array}}^{30} {\beta }_tBuye{r}_h*{D}_t + {\partial }_h + {\varepsilon }_{th}, \end{equation*}

where the outcome of interest$,\ {Y}_{th}$, is the monthly average purchase (measured in grams) of packaged noodles in period $t$ by household $h$. $t$ indicates each month in our data period. It is set at 0 for May 2015 when the Scandal first began and hence ${- 23} \le t \le 30$. $\alpha $ is the constant term. ${D}_t\ $are dummy variables for each month $t$. We use the month just before the start of the Scandal (i.e. $t = - 1$) as the reference month and hence omit the corresponding variable (${D}_{t = - 1}$). These time dummies capture the effects of other time-variant events that might have affected the noodle purchases of both household groups, such as changes in prices or stock level of noodles and other foods, as well as other macroeconomic events in India. $Buye{r}_h$ takes the value of 1 for the buyer group which bought Maggi noodles before the Scandal and 0 for the comparison group (non-buyer). ${\beta }_t\ $give the event study estimates, which are the coefficient of the interaction terms between the buyer status and time dummies. We again exclude ${\beta }_{t = - 1}$ to normalize these differences to the period just before the onset of the Scandal. ${\partial }_h$ is household fixed effect. Since the buyer status does not change over time, $\gamma $ will be absorbed by the household fixed effect in the estimation. ${\varepsilon }_{th}$ is the random error clustered at household level to account for the correlation in purchase pattern of households.

Given that our comparison group are households who did not purchase any Maggi noodles prior to May 2015, it is not feasible to directly estimate the Scandal impacts on Maggi noodle purchases. As an alternative, we use two outcome measures—(i) purchases of non-Maggi packaged noodles only and (ii) purchases of all packaged noodles. The first outcome allows us to study if the buyer households substituted Maggi noodles with other noodles due to the Scandal and continued to buy them after Maggi noodles returned to the market, in other words, the spillover effect of the Scandal. The second outcome illustrates how the overall packaged noodle purchases of the buyer households changed in response to the Scandal and to what extent these changes remained in the post-Scandal period. By comparing both outcomes, we can infer the dynamics of the disproportionate changes in Maggi noodle purchases across the buyer groups.

Among the event study estimates, ${\beta }_{t < - 1}$ give the pre-Scandal differences in purchases between the buyer and comparison groups. ${\beta }_{t \ge 0}$ illustrate the changes in purchases of noodles among the buyer groups over the comparison group from May 2015 onward after controlling for time-varying confounding events through ${\tau }_t$ and time-invariant household heterogeneity through ${\partial }_h$. It should be noted that ${\beta }_{1 \le t \le 5}$ can be driven by both the decline in supply shock and the reputation effects of the Scandal on Maggi noodles as the sale of Maggi noodles was banned nationwide from June 2015 to November 2015. Since this ban occurred rather quickly after the initial news reports, it is not possible to distinguish these two mechanisms with the current data available. ${\beta }_{t > 5}$ capture the long-term changes in noodles purchase that were driven by the reputation effects as the supply shock disappeared with Maggi noodles back on shelves.

### 5.2 Threat to identification

As in any non-randomized studies, selection bias is one of the key threats to identification. The event study estimates may be biased by the heterogeneity between households in the buyer groups and the ones in the comparison group. The inclusion of household fixed effects accounts for any differences in their noodle purchases driven by household -specific factors that remained stable during the data period. This removes any bias from time-invariant unobservable heterogeneity between the two groups. Additionally, we apply an inverse propensity weighting to improve the comparability between the buyer and comparison groups. This approach ensures that they are more likely to face similar time-varying trend changes. The weighting approach adjusts the event study estimates using the probability that a household would have been assigned to a buyer category. This is achieved by upward weighting the outcome levels for households from the comparison group who are under-represented and placing lower weight on those who are over-represented (Rosenbaum & Rubin 1983). For each buyer-comparison group pair, we estimate a binominal probit regression to predict probability of the household being in the corresponding buyer group or the comparison group (non-buyer) based on observed predictors, including state dummies, household composition, household size, and socio-economic classes. The weights are then generated using the inverse of the estimated propensity score.^11^

However, there remains concerns over the potential selection bias from unobserved time-varying heterogeneity that correlate systematically with the buyer status. In the difference-in-differences framework, this bias is minimal if the noodle purchases by the buyer and comparison groups would have evolved similarly in the absence of the Scandal. Under this parallel trend assumption, any post-event changes in the outcomes for the buyer group beyond the common trend could be identified as the true consumer response to the Scandal. While this assumption is not testable, we can identify signs of potential violation of this assumption through a pre-trend test using the event study estimates for periods prior to the Scandal (${\beta }_{t < 0}$). If they are statistically insignificant, it signals that both groups shared a common trend prior to the Scandal and thus the parallel trend assumption is unlikely to be violated.

Recent difference-in-differences literature has highlighted the limitation of relying on parallel trend assumption to infer the validity of the estimates (Roth et al. 2022). In particular, the formal inference test proposed by Rambachan and Roth (2020) has been increasingly used as a sensitivity check against this assumption (e.g. Ang, 2020; Dustmann et al. 2021; Rose, 2021). Following their approach, we examine the potential impact of differential pre-trends of noodle purchases among the buyer and comparison groups on our key estimates. This is done by allowing any pre-event trends to continue and additionally limiting the degree to which the trends in the post-event period can deviate from those in the pre-event period.^12^ These test results give an indication on whether our main findings hold under the potential bias from the time-varying heterogeneity among the buyer and comparison groups, which would be particularly important in cases where the parallel trend prior to the Scandal is less convincing.

To further ensure validity of our empirical approach, we conduct placebo tests by repeating our analysis on purchases of salty snacks, which include potato chips and traditional snacks, and crackers. These products are similar to packaged noodles in terms of shelf life and the degree of food processing. One potential concern over our findings is that they were driven by changes in food preferences that were specific to the buyer groups but unrelated to the Scandal. For example, if the buyers had become more health conscious, they could have reduced their purchases of highly processed foods and hence Maggi noodles, even if the Scandal did not occur. We compare the purchases of these products between the buyer and the comparison groups and investigate if the estimates we observe from our main analysis are specific to noodles. A null result on the purchases of salty snacks and crackers, which were not implicated by the Scandal, will suggest that our findings are unlikely to be driven by the selection bias from the buyer status assignment.

Considering the large market share of Maggi noodles prior to the Scandal, the nationwide ban of these products from May to November 2015 was likely to substantially reduce the supply of packaged noodles, which might have increased prices and subsequently led to a further decline in noodle purchases. One limitation of our purchase data is that it does not hold any information on product prices. As a result, we rely on the difference-in-differences approach to control for the price changes in packaged noodles that should be common to both buyer and comparison groups. To reassure that our main findings are not driven by price changes, we perform a synthetic control analysis on the noodle prices in India using the product-level monthly wholesale price index (WPI) from February 2013 to December 2017.^13^ The method, formalized by Abadie, Diamond, and Hainmueller (2010), uses a weighted average of outcomes from the donor pool as the counterfactual to account for time-varying unobservables. The optimal weights are selected by minimizing differences in predictors between noodles and the synthetic control. We use the WPI of all other food items provided as the donor pool and their pre-Scandal levels as predictors. The difference between the WPI of noodles and its synthetic control from May 2015 would indicate any abnormal price changes in noodles that might have impacted our findings.^14^

## 6 Results

### 6.1 Main findings

Our key coefficients of interest are ${\beta }_{t \ge 0}$, the event study estimates since the beginning of the Scandal in May 2015. To aid our understanding, we group these estimates into three groups based on the time periods: (i) ${\beta }_{0 \le t \le 5}\ $indicate the immediate responses to the Scandal, up to the return of Maggi noodles to the market in November 2015. (ii) ${\beta }_{6 \le t \le 18}$ capture the 1-year post Scandal response. (iii) ${\beta }_{19 \le t \le 30}$ provide the post-Scandal response in the second year after Maggi noodles were back on shelves. For each group, we present the average estimate per month in Table 2. Using the non-buyer as the comparison group, columns 1–3 give the unweighted event study estimates on the noodle purchases of monthly, frequent, and infrequent buyers, respectively. In columns 4–6, inverse propensity weighting is applied to improve the comparability between the buyer and comparison groups. Given the concern over selection bias, we focus our discussion on the weighted results. Despite the small differences in magnitude, their sign and statistical significance are largely consistent with the unweighted estimates.

**Table 2.** Changes in noodle purchases in response to the Scandal (in grams/month).

	Unweighted			Inverse propensity weighting		
Buyer group	Monthly buyer	Frequent buyer	Infrequent buyer	Monthly buyer	Frequent buyer	Infrequent buyer
Comparison group	Non-buyer			Non-buyer		
	(1)	(2)	(3)	(4)	(5)	(6)
*Panel A: Non Maggi Noodles (Reference period: April 2015)*						
Immediate response	62.396^***^	18.861^***^	−8.058^***^	64.001^***^	22.720^***^	−8.932^***^
(May 2015–Nov 2015)	(5.397)	(3.468)	(3.237)	(5.595)	(9.356)	(3.768)
1-year post response	72.969^***^	36.672^***^	2.933	66.660^***^	36.130^***^	2.919
(Dec 2015–Nov 2016)	(5.580)	(3.378)	(3.147)	(5.561)	(6.794)	(3.153)
2-year post response	35.110^***^	14.466^***^	−4.822	31.853^***^	15.625	−4.418
(Dec 2016 -Nov 2017)	(5.397)	(3.577)	(3.371)	(5.729)	(9.143)	(3.634)
*Panel B: All noodles (Reference period: April 2015)*						
Immediate response	–355.051^***^	–214.231^***^	–68.169^***^	–361.683^***^	–214.541^***^	–70.180^***^
(May 2015–Nov 2015)	(12.739)	(4.146)	(3.387)	(13.374)	(9.653)	(3.949)
1-year post response	–148.871^***^	–111.824^***^	–42.385^***^	–143.852^***^	–115.186^***^	–43.188^***^
(Dec 2015–Nov 2016)	(12.075)	(4.060)	(3.342)	(12.159)	(6.379)	(3.269)
2-year post response	–119.481^***^	–85.221^***^	–36.703^***^	–117.681^***^	–95.579^***^	–38.102^***^
(Dec 2016–Nov 2017)	(12.957)	(4.417)	(3.699)	(14.364)	(12.822)	(3.945)
Household fixed effect	Yes	Yes	Yes	Yes	Yes	Yes
N	191,160	1,137,672	1,420,848	191,160	1,137,672	1,420,848

Note: Columns 4–6 present the average event study estimates within the corresponding period shown in Figs. 3 and 4. Buyer groups are defined based on the number of months they purchased Maggi noodles during the 24 months prior to May 2015: 24 months for Monthly buyer; 13–23 months for Frequent buyer; 1–12 months for Infrequent buyer. Comparsion households are those who did not buy any Maggi noodles for 2 years prior to May 2015. Robust standard errors clustered at household level.

* *P* < 0.1.

** *P* < 0.05.

*** *P* < 0.01.

Panel A in Table 2 provides evidence of the spillover effect of the Scandal on non-Maggi noodle purchases. The direction of this effect differs across households with different Maggi noodle purchasing habits prior to the Scandal. During the Scandal, while the monthly and frequent buyers increased their purchases of non-Maggi noodles by 64g per month (i.e. immediate response in column 4) and 22.7g per month (column 5) relative to the comparison group, the infrequent buyer reduced their purchases of these noodles by 8.9g per month (column 6). The increased purchases of non-Maggi noodles remained significant among the monthly and frequent buyers in the first year after Maggi noodles returned to the market (i.e. 1 year post-Scandal response). However, the increase in non-Maggi noodles purchases by the infrequent buyer dissipated during the same period. The 2-year post-Scandal response is only found statistically significant among the monthly buyer. Relative to the comparison group, they increased their purchases of non-Maggi noodles by 66.7g and 31.8g per month in the first and second years after the return of Maggi noodles respectively. Overall, it appears that the spillover effect of the Scandal is more persistent and positive among households with stronger purchasing habit of Maggi noodles.

The average event study estimates on all noodle purchases are given in panel B of Table 2. Column 4 shows that monthly buyer reduced their purchases of packaged noodles by 361.7g per month during the Scandal (i.e. immediate response) relative to the comparison group while the frequent and infrequent buyers lowered their all noodle purchases by 214.5g (column 5) and 70.2g (column 6) per month, respectively. This shows that the magnitude of the effect declines in absolute terms with the purchase frequency. For all groups, the reduction in purchases of all noodles gradually becomes smaller over time as Maggi returned to the market but remained statistically significant up to the end of 2017.^15^

**Figure 3.**
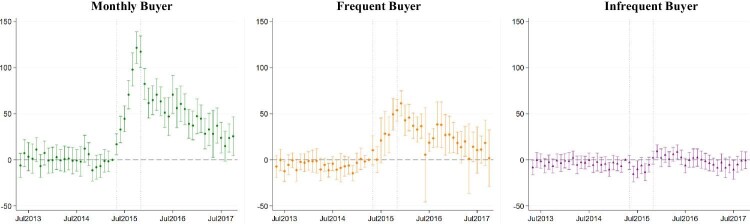
Event study estimates on non-Maggi noodle purchases (in grams) across buyer groups. Note: This figure shows the event study estimates and the 95 per cent confidence interval from estimation of Equation 1 with inverse propensity weighting on non-Maggi noodle purchases. Standarad errors clustered at household level. Buyer groups are defined based on the number of months they purchased Maggi noodles during the 24 months prior to May 2015: 24 months for Monthly buyer; 13–23 months for Frequent buyer; 1–12 months for Infrequent buyer. Comparsion households are those who did not buy any Maggi noodles 2 years prior to May 2015. Dotted lines indicate the scandal period from May 2015 to November 2015.

**Figure 4.**
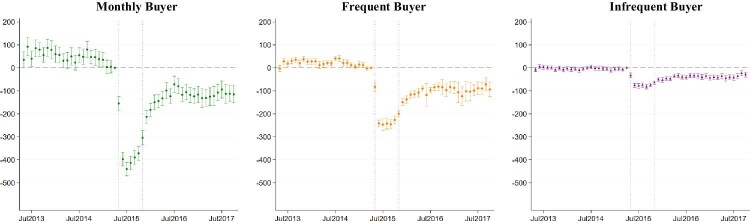
Event study estimates on all noodle purchases (in grams) across buyer groups. Note: This figure shows the event study estimates and the 95 per cent confidence interval from estimation of Equation 1 with inverse propensity weighting on all noodle purchases. Standarad errors clustered at household level. Buyer groups are defined based on the number of months they purchased Maggi noodles during the 24 months prior to May 2015: 24 months for Monthly buyer; 13–23 months for Frequent buyer; 1–12 months for Infrequent buyer. Comparsion households are those who did not buy any Maggi noodles 2 years prior to May 2015. Dotted lines indicate the scandal period from May 2015 to November 2015.

Additionally, we plot all weighted estimates of ${\beta }_t$ on purchases of non-Maggi noodles and all noodles in Figs. 3 and 4, respectively. These figures not only allow visual analyses of the dynamics of the consumer response to the Scandal but also inspection of the pre-Scandal differences in noodle purchases between the buyer and the comparison groups. Fig. 3 shows that the event study estimates for non-Maggi noodle purchases were statistically insignificant prior to May 2015 for all three buyer groups. The monthly buyer started to increase their purchases of non-Maggi noodles in May 2015 and this continued to rise until Maggi noodles were back on shelves in November 2015. The spillover to non-Maggi noodles persisted over the two years after the return of Maggi noodles although its magnitude declined over time. Consumer response in similar pattern but in smaller magnitude can also be seen among the frequent buyer. The spillover effect on their purchases of non-Maggi noodles is; however, less persistent as most estimates become statistically insignificant in 2017. Unlike the other two buyer groups, there is some evidence that the infrequent buyer reduced their purchases of non-Maggi noodles temporarily during the Scandal, suggesting that they may have associated the negative food safety information with other noodle brands. The absence of significant pre-Scandal event study estimates suggests that the parallel trend assumption is unlikely to be violated for purchases of non-Maggi noodles.

Next, we turn to the event study estimates on all noodle purchases in Fig. 4. All three buyer groups reduced their purchases in May 2015 when the Scandal first started. A further decline can be seen in June when Maggi noodles were banned across India. Consistent with the results in Table 2, this reduction in all noodle purchases persisted over the two years after the return of Maggi noodles. While there is no evidence for differential pre-trend between the infrequent buyer and the comparison group, some of the estimates prior to May 2015 were statistically significant and positive for monthly and frequent buyers. This gives rise to the plausibility of a declining trend in the difference of all noodle purchases between these two buyer groups and the comparison group, making the parallel trend assumption less convincing in these cases. This also suggests that monthly and frequent buyers may have reduced their purchases of Maggi noodles prior to the Scandal as no significant estimates was observed in non-Maggi noodle purchases (Fig. 3) prior to May 2015. However, it should be noted that these differential pre-trends appear to have dissipated in February 2015. Furthermore, given that the event study estimates were much larger in absolute value during the Scandal period (i.e. May 2015–November 2015), it is unlikely that the dramatic declines displayed in Fig. 4 (and the estimates in Table 2) were solely driven by the potential differential underlying trends. As a robustness check, we empirically test whether our results would be invalid if these pre-existing trends were to continue after February 2015.

### 6.2 Sensitivity checks for the parallel trend assumption

We apply Rambachan and Roth (2020)’s approach to formally examine the sensitivity of our estimates presented in Table 2 to violations of parallel trend assumption. This includes assessment of the potential downward bias in estimates should the trends in all noodle purchases by monthly and frequent buyers prior to February 2015 continue.

The results presented in Appendix B illustrate that our main findings hold under prevailing non-parallel trends and are unlikely to be driven by the selection bias from time-varying heterogeneity. We continue to observe an immediate decline in all noodle purchases by the three buyer groups relative to the comparison group during the Scandal period. This reduction in purchases persisted over the two years after Maggi noodles were back on shelves. There remains strong evidence in support of the positive spillover effects to purchases of non-Maggi noodles among the monthly and frequent buyers.

### 6.3 Placebo tests

We repeat the event study analysis on the purchases of crackers and salty snacks for each buyer group as a placebo test. If our findings on noodle purchases were driven by unobserved time-varying heterogeneity among the buyer and comparison groups such as increasing awareness of healthier food choices, we should observe significant changes in the purchases of these foods too. The placebo estimates for purchases of crackers and salty snacks are presented in figures C1 and C2, respectively. Similar to all noodle purchases (Fig. 4), some placebo estimates prior to February 2015 are positive and statistically significant for the monthly and frequent buyers. However, we find no evidence for any significant changes in purchases of these snacks by any of the three buyer groups since the start of the Scandal (May 2015). These placebo tests suggest that the significant changes in purchases are unique to packaged noodles and hence unlikely to be driven by time-varying unobservables that are correlated with household food purchases.

### 6.4 Synthetic control analysis on the WPI of noodles

**Figure 5.**
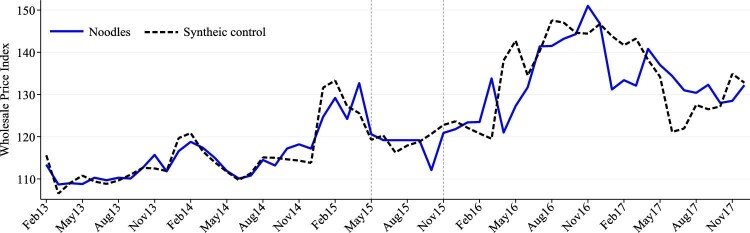
WPI of noodles in India and its synthetic control, January 2012–December 2017. Note: Dotted lines indicate the Scandal period from May to November 2015.

Finally, we address the concerns over possible Scandal-induced price changes through a synthetic control analysis of the WPI of noodles. Fig. 5 shows that the synthetic control (dash line) closely tracks the WPI of noodles (solid line) in India during the pre-Scandal period. They were initially at a similar level in June 2015 when Maggi noodles were removed from the market, suggesting that the ban did not cause a substantial increase in the price of noodles. The WPI of noodles remained stable until September 2015 while its synthetic control decreased slightly and then bounced back. The small gap between the two lines suggests that noodles may have become relatively more expensive during this period. However, this gap was only temporary as the WPI of noodles decreased in October 2015 and went back up to a level similar to its synthetic control in November 2015. Overall, there is no evidence for a substantial increase in noodle prices after the ban of Maggi noodles that could have explained the decline in purchases of all noodles found in this paper.

## 7 Discussion and conclusion

The Maggi noodles scandal is a clear example of how consumers are prepared to make persistent changes in their purchase patterns in response to food safety concerns even if that food product is an integral part of their food culture. During the Scandal period, households who had bought Maggi noodles monthly prior to May 2015 decreased their purchases of all packaged noodles significantly relative to households who did not buy any. Reductions in all noodle purchases of smaller magnitude are also observed among households with lower purchase frequency of Maggi noodles. These Scandal effects remained statistically significant over the two years after Maggi noodles were back on shelves. We also find evidence that the purchases of non-Maggi noodles increased among the monthly and frequent buyers since the start of the Scandal but decreased among the infrequent buyer. While the latter negative spillover effect dissipated after the return of Maggi noodles, the positive spillover effects among households with more regular purchasing habits remained statistically significant in the year after. These results are unique to packaged noodles purchases and robust to alternative assumptions of pre-Scandal trend in purchases.

Taken together the findings on the two main outcomes, we find strong evidence that the Scandal had a negative and persistent impact on the purchases of Maggi noodles across all buyer groups. In response to the food safety concerns, monthly and frequent buyers reduced all noodle purchases while buying more non-Maggi noodles than before, indicating a shift in their demand away from Maggi noodles. For infrequent buyer, their purchases of Maggi noodles also reduced during the Scandal period as the reduction in purchases of all noodles outweighed the reduction in purchases of non-Maggi noodles. To a large extent, these purchase changes persisted after Maggi noodles returned to the market as the reduction in all noodle purchases remained statistically significant and there was no evidence of a decline in non-Maggi noodle purchases.

One caveat is that this paper uses households who did not buy any Maggi noodles in the 24 months prior to the Scandal as the comparison group. Our results might be biased if these comparison households become concerned about consuming packaged noodles due to the Scandal and thus reduced their purchases of other packaged noodles. This implies that changes in noodle purchases across buyer groups could be underestimated and hence our results could be viewed as the lower bound of non-buyer households. However, this downward bias is likely to be minimal given that some of them started to purchase Maggi noodles after November 2015 when Maggi noodles were back on shelves as indicated in Fig. 1.

Our results provide three policy lessons. First, this paper finds evidence on the persistence of consumer response to food safety concerns. Most studies on food safety crises analyze the changes in purchases in the short run. One of the more persistent changes can be seen in the BSE outbreak in Japan in which the negative impact on meat purchases diminished gradually over eight months although similar persistent changes are not found in the case of bird flu scares (Ishida et al. 2010). This paper reveals that the Maggi noodles scandal had a persistent negative effect on all noodle purchases among households who used to buy Maggi noodles, compared to those who never bought Maggi noodles. We also show that the Scandal had a positive and long-term spillover effect on the purchases of non-Maggi noodles by households with regular purchasing habits but not infrequent buyer. This illustrates that food safety concerns could have more profound effects by opening a window for other brands to capture market share and build brand loyalty.

Second, our findings illustrate that the type of market matters when evaluating the effect of safety concerns on collective reputation. Our results on the spillover effects to other noodles brands are aligned with Bakhtavoryan et al. (2014) which also find that the food recall of a particular brand bought positive consumer response to brands that were not recalled. On the other hand, studies that looked into recalls of branded toys, automobiles, and drugs find negative spillover effects (Cawley & Rizzo 2008; Freedman et al. 2012; Bachmann et al. 2019). This indicates that consumers may react to product safety information differently depending on the product nature. In the case of packaged noodles, frequent customers in India did not stigmatize alike food products by different brands and substituted some of their purchases of Maggi noodles with non-Maggi noodles. This positive spillover effect may encourage firms to invest in product brandings to differentiate their products from others, which would serve as a signal to consumers about the safety and quality of the products as well as an insulation to any negative information about other products in the market. Further investigation into the reasons behind the heterogeneity in spillover effects across product recalls would provide deeper insights into the benefits of branding and the likelihood of collective reputation in different industries.

Lastly, the persisting changes in the packaged noodle purchase pattern caused by the Scandal demonstrate that food producers failing to meet food safety regulations could potentially face huge loses in their consumer base. The shift from Maggi noodles to other brands gives support for the rising awareness for better and safer food in LMICs. Additionally, our results reveal that brand commitment does not necessarily insulate food producers from the reputation effect of recalls. The long-term loss in purchases, as demonstrated in this paper, combined with large costs of marketing campaigns to address the reputational concerns, provide strong incentive for food companies to comply with government regulations and ensure safety of their products. Governments could leverage this reputation effect to introduce comprehensive regulations to incentivize firms to provide foods that are safe for human health.

## Supplementary Material

qoac025_Supplemental_File

## Data Availability

Purchase data, were provided by “Kantar-Worldpanel Division, India”. The terms of our data agreement with Kantar mean that we cannot share these data.

## Footnotes

1 They are instant noodles that can be cooked in a few minutes as a snack or part of a meal. They are popular comfort foods among all economic groups in India (Baviskar 2018). In 2014, it was estimated that over 400,000 tons of Maggi noodles were consumed by Indians, making it the leading instant noodle brand (Fortune 2016).

2 In addition to consumer responses, empirical research from developed countries has investigated the effects of food recalls on stock market returns (Salin & Hooker 2001; Thomsen & McKenzie 2001; ; Seo et al. 2013; Pozo & Schroeder 2016), product price movement (Carter & Smith 2007; Moon & Tonsor 2020); future market prices (Lusk & Schroeder 2002; Schlenker & Villas-Boas 2009; Moghadam et al. 2013; Houser et al. 2019), and post-recall food safety performance (Ollinger & Bovay 2020; Ollinger & Houser 2020).

3 A related strand of literature to food safety recalls is the consumer responses to negative media coverage of food products in the US (e.g. Piggott and Marsh, 2004; Mazzocchi, 2006; Yadavalli and Jones, 2014). Pritchett et al. (2007) show that media coverage of BSE cases in Canada and the US negatively impacted the US demand for ground beef and chuck roasts, while positively impacting center-cut pork chop demand. The negative demand effects of the BSE outbreaks have also been studied in other countries such as Japan ( Jin & Koo 2003; Ishida et al. 2010), the United Kingdom (Burton & Young 1996) and Canada ([Bibr bib21][Bibr bib22]).

4 Kong et al. (2019) found that food product recalls result in significant negative abnormal returns for listed firms in China. To our best knowledge, it is the only empirical study which examined the financial effects of food recalls in LMICs.

5 During the ban of Maggi noodles, Sunfeast Noodles, the second largest noodle brand in India, rolled out a marketing campaign focusing on the quality and safety of their products (The Economic Times 2015).

6 The data period covers two years prior to the start of the Scandal and two years after the return of Maggi noodles to the market (55 months in total).

7 Since Maggi noodles require a certain level of cooking, they are commonly consumed at home. The lack of data on purchases made for consumption away from home is unlikely to cause significant measurement errors.

8 These weights are computed by the ‘Kantar—Worldpanel Division, India’ to ensure that total market estimates are as accurate as possible.

9 For a consistent household panel, we only consider households who reported purchases of any processed foods for over 53 months during the 55 months data period. In addition, households with more than a 2 month gap in reporting were excluded.

10 Purchase frequency is commonly used in the marketing literature to measure behavioral loyalty (Sharp & Sharp 1997; Dawes et al. 2015). It is one of the key parameters in the Dirichlet model, which is a well-established marketing model to describe brand choice of consumer product (Dawes et al. 2015).

11 The probit regression and balancing results are provided in Appendix A.

12 This approach is explained in greater detail in Appendix B.

13 The WPI data was obtained from the Office of the Economic Advisor of the Government of India https://eaindustry.nic.in/download_data_1112.asp.

14 Full specification and the list of food items in the donor pool are provided in the Appendix D.

15 In Appendix E, we present the average event study estimates stratified by total purchases of Maggi noodles prior to the Scandal. Within each buyer group, we compute the average total purchase of Maggi noodles during the 24 months prior to May 2015, which is then used to further classify households into above and below average purchase of Maggi noodles. The stratified results are consistent with the main findings, showing that consumers reduced the quantity they purchased after the Scandal. Within each buyer group, the reductions in all packaged noodle purchases are found to be higher among the subgroup with above average pre-Scandal purchases of Maggi noodles. We continue to observe positive spillover effects over non-Maggi noodle purchases among both subgroups of monthly buyer as well as frequent buyer, which are typically smaller than the negative effects on their total noodle purchases (in absolute term).
