# Supplementary material for: Persistent consumer response to a nationwide food safety recall in urban India
Source: Q Open. 2022 Sep 20;2(2):qoac025. doi: 10.1093/qopen/qoac025 (PMC9537021; doi:10.1093/qopen/qoac025)
Supplement: qoac025_Supplemental_File [file qoac025_supplemental_file.docx]

**Online supplementary materials**

1. **Inverse propensity weighting**

Table A1 Probit regression on probability of belonging to the respective buyer group over the comparison group

|  | Probability of belonging to the respective buyer group | | |
| --- | --- | --- | --- |
|  | Infrequent buyer | Frequent buyer | Monthly buyer |
| VARIABLES | (1) | (2) | (3) |
| Socio-economic class (Reference group=lower class) |  |  |  |
| Upper class | 0.529*** | 1.265*** | 1.708*** |
|  | (0.0366) | (0.0432) | (0.105) |
| Upper middle class | 0.396*** | 0.783*** | 0.820*** |
|  | (0.0351) | (0.0426) | (0.105) |
| Middle class | 0.226*** | 0.435*** | 0.408*** |
|  | (0.0343) | (0.0426) | (0.109) |
|  |  |  |  |
| Household size | 0.00159 | 0.00298 | 0.00550 |
|  | (0.00747) | (0.00830) | (0.0184) |
| Presence of children in the following age groups in the household | | | |
| Infant | 0.0972 | 0.221*** | 0.302* |
|  | (0.0743) | (0.0811) | (0.175) |
| 1 year old | 0.0314 | 0.161** | 0.391*** |
|  | (0.0617) | (0.0678) | (0.141) |
| 2– 4 years old | 0.0711* | 0.257*** | 0.254*** |
|  | (0.0394) | (0.0426) | (0.0927) |
| 5 – 9 years old | 0.135*** | 0.292*** | 0.364*** |
|  | (0.0317) | (0.0342) | (0.0762) |
| 10 – 14 years old | 0.147*** | 0.327*** | 0.440*** |
|  | (0.0276) | (0.0303) | (0.0677) |
| 15 – 17 years old | 0.0767*** | 0.185*** | 0.289*** |
|  | (0.0290) | (0.0324) | (0.0731) |
| Constant | 1.125*** | 0.538*** | -1.238*** |
|  | (0.0734) | (0.0811) | (0.162) |
|  |  |  |  |
| Observations | 26,312 | 21,068 | 3,540 |

Note: Buyer groups are defined based on the number of months they purchased Maggi noodles during the 24 months prior to May 2015: 24 months for Monthly buyer; 13-23 months for Frequent buyer; 1-12 months for Infrequent buyer. Comparsion households are those who did not buy any Maggi noodles 2 years prior to May 2015. Standard errors in parentheses ***p<0.01, **p<0.05 *p<0.1

Table A2 Summary statistics with inverse propensity weighting.

|  | **Monthly Buyer** | |  | **Frequent Buyer** | |  | **Infrequent Buyer** | |
| --- | --- | --- | --- | --- | --- | --- | --- | --- |
|  | Mean of buyer group* | Weighted mean of comparison group | | Mean of buyer group* | Weighted mean of comparison group | | Mean of buyer group* | Weighted mean of comparison group |
| Household size | 5.208 | 4.765 |  | 4.931 | 5.371 |  | 4.650 | 4.635 |
| Presence of children in the following age groups in the household | | | | | | | | |
| Infant | 0.033 | 0.025 |  | 0.029 | 0.026 |  | 0.025 | 0.025 |
| 1 year old | 0.049 | 0.047 |  | 0.043 | 0.052 |  | 0.038 | 0.040 |
| 2– 4 years old | 0.152 | 0.129 |  | 0.152 | 0.208 |  | 0.120 | 0.120 |
| 5 – 9 years old | 0.290 | 0.207 |  | 0.266 | 0.319 |  | 0.218 | 0.216 |
| 10 – 14 years old | 0.403 | 0.311 |  | 0.349 | 0.415 |  | 0.291 | 0.281 |
| 15 – 17 years old | 0.306 | 0.220 |  | 0.254 | 0.219 |  | 0.231 | 0.224 |
| Social economics class | |  |  |  |  |  |  |  |
| Upper class | 0.612 | 0.312 |  | 0.476 | 0.416 |  | 0.298 | 0.285 |
| Upper middle class | 0.232 | 0.264 |  | 0.284 | 0.324 |  | 0.309 | 0.307 |
| Middle class | 0.108 | 0.267 |  | 0.179 | 0.179 |  | 0.274 | 0.278 |
| Lower class | 0.048 | 0.158 |  | 0.061 | 0.082 |  | 0.119 | 0.130 |
| States |  |  |  |  |  |  |  |  |
| Delhi | 0.225 | 0.065 |  | 0.059 | 0.049 |  | 0.023 | 0.023 |
| Jharkhand | 0.052 | 0.028 |  | 0.085 | 0.066 |  | 0.050 | 0.048 |
| Andhra Pradesh | 0.003 | 0.072 |  | 0.040 | 0.043 |  | 0.092 | 0.095 |
| Maharashtra | 0.109 | 0.098 |  | 0.126 | 0.135 |  | 0.118 | 0.121 |
| Punjab/Haryana | 0.218 | 0.101 |  | 0.149 | 0.221 |  | 0.027 | 0.025 |
| West Bengal | 0.050 | 0.103 |  | 0.064 | 0.059 |  | 0.067 | 0.071 |
| Gujarat | 0.023 | 0.062 |  | 0.044 | 0.038 |  | 0.069 | 0.068 |
| Karnataka | 0.009 | 0.048 |  | 0.033 | 0.036 |  | 0.074 | 0.076 |
| Kerala | 0.007 | 0.038 |  | 0.030 | 0.032 |  | 0.067 | 0.067 |
| Rajasthan | 0.023 | 0.027 |  | 0.028 | 0.020 |  | 0.037 | 0.034 |
| Orissa | 0.005 | 0.009 |  | 0.035 | 0.022 |  | 0.037 | 0.027 |
| Madhya Pradesh | 0.039 | 0.109 |  | 0.065 | 0.072 |  | 0.091 | 0.099 |
| Uttar Pradesh | 0.156 | 0.136 |  | 0.130 | 0.124 |  | 0.097 | 0.102 |
| Tamil Nadu | 0.012 | 0.072 |  | 0.057 | 0.049 |  | 0.114 | 0.109 |
| Bihar | 0.069 | 0.032 |  | 0.053 | 0.035 |  | 0.036 | 0.035 |

Note: * indicates the unweighted mean for each buyer group. Buyer groups are defined based on the number of months they purchased Maggi noodles during the 24 months prior to May 2015: 24 months for Monthly buyer; 13-23 months for Frequent buyer; 1-12 months for Infrequent buyer. Comparsion groups are those households who did not buy any Maggi noodles 2 years prior to May 2015.

1. **Sensitivity checks for violations in the parallel trend assumption**

We apply Rambachana and Roth (2022) approach to formally assess the robustness of our results to alternative assumption about different trends among treatment and control group. Recent economic studies employ this approach to illustrate their results are robust to linear deviation from the pre-treatment difference in trends (“pre-trend) across treatment and control groups. This is particularly important for our findings on the purchase response of the monthly buyer due to the declining pre-trend shown in Figure 3. This implies a potential violation of the parallel trend assumption.

Based on Rambachana and Roth (2022), we impose restrictions on the degree to which parallel trend can fail in the post-treatment period and report the confidence interval of the estimates presented in table 2 in the main paper. The possible differences in trends between the treated and control groups are bounded using the following formula^[[1]](#footnote-2)^:

$$\Delta^{SD}\left( M \right)= \left\{ \delta:\left| {(\delta}_{t}-\delta_{t-1} \right)-{(\delta}_{t-1}-\delta_{t-2})|\leq M \right\}$$

Where $\delta_{t}$represnets the difference in trends between the buyer and control groups at time $t$. For the choice of M=0, it assumes that the violation of parallel trends to be linear, following the same magnitude. M>0 relaxes this assumption and introduces changes in the slope of the underlying period to be no more than M between consecutive post-treatment periods. Similar to Dustmann et al., (2021), we set M at a value that allows a 20% deviation from the linear trend line of the pre-Scandal purchases differences between the buyer and the non-buyer groups. It should be noted that it is a conservative approach as it accumulates to 80% deviation after 4 months (Dustmann et al. 2021). For example, a value of M =0.053 is used to examine the robustness of the Scandal effects on monthly buyer’s non-Maggi noodle purchases of as the slope of the pre-Scandal purchase differences is 0.264. We summarise the values of M used for two outcomes of interest in the table below.

Table B1 Values of M used to assess the parallel trend assumption

|  | **Non-Maggi noodles** | | **All noodles** | |
| --- | --- | --- | --- | --- |
|  | S | M | S | M |
| Monthly buyer | 0.264 | 0.053 | 2.100 | 0.420 |
| Frequent buyer | 0.025 | 0.005 | 0.523 | 0.105 |
| Infrequent buyer | 0.099 | 0.020 | 0.222 | 0.044 |

Note: S= Slope of pre-Scandal differences the purchase between the corresponding buyer and non-buyer groups (in absolute value). M is the maximum violation of the differential trend considered in the sensitivity check.

Figure B1 shows the estimated post-Scandal changes in the noodle purchases of monthly buyers under relaxation of parallel trend assumption. In each plot, the first (dark blue) and fifth (dark red) markers indicate the 95% confidence intervals of the main estimates (under the parallel trend assumption) on purchases of non-Maggi and all noodles respectively. The rest of thin markers represent the 95% fixed length confidence intervals of the estimated consumer response to the Scandal for non-Maggi (blue) and all noodles purchases (red) under different magnitudes of parallel trend violation, which is indicated by $M$. $M=0$ assumes that the noodle purchases made by both groups would have followed a linear trend based on the pre-Scandal trend and $M=.042$ allows for a 20% deviation in the slope of each outcome each month. Relaxing the parallel trend assumption has minimal impact on the estimates of the immediate responses to the Scandal. Similar to other studies applied this method, our results continue to hold under linear violation of parallel trend but become increasingly difficult to conclude when M grows larger.^[[2]](#footnote-3)^ Monthly buyer households still display significantly lower purchases of all noodles in the year after the Scandal although the magnitude of the decline decreases when M>0. The 2-year post responses of monthly buyer to the Scandal are more sensitive to non-linear deviations from parallel trends. This is not surprising as a 20% deviation per month accumulates to over 300% deviation after 17 months.

Figure B1 Parallel trend test of noodle purchases for **monthly** **buyer**

Note: Each plot presents the estimated average monthly consumer response to the Scandal based on alternative assumptions about the evolution of noodle purchases among monthly buyer versus comparison group in the absence of the Scandal. The first (dark blue) and the fifth (dark red) lines represent the 95% confidence intervals for the original estimates for non-Maggi and all noodle purchases respectively (i.e. column 4 in table 2), which are based on the parallel trend assumption. Additional thin lines (blue and red) are the fixed length confidence intervals when allowing for violation of parallel trends of up to 𝑀, which is the maximum possible change in the slope of an underlying purchase trend between each month. Hence, $M=0$ allows for linear deviation.

For frequent and infrequent buyer, their purchase responses to the Scandal are robust to linear violations of parallel trend assumption (Figures B2 and B3 respectively). Similar to monthly buyer, the estimated 2-year post-Scandal responses of frequent buyer is less robust when M>0. These test results illustrate that our main findings hold under non-parallel prevailing trends and are unlikely to be driven by the selection bias from time-varying heterogeneity. We continue to observe immediate declines in all noodle purchases by the three buyer groups relative to the comparison group during the Scandal period. This reduction in purchases remains statistically significant over the year after Maggi noodles were back on shelves. There remains strong evidence in support of the positive spillover effects over purchases of non-Maggi noodles among the monthly and frequent buyers.

Figure B2 Parallel trend test of noodle purchases for **frequent** **buyer**

Note: Each plot presents the estimated average monthly consumer response to the Scandal based on alternative assumptions about the evolution of noodle purchases among frequent buyer versus comparison group in the absence of the Scandal. The first (dark blue) and the fifth (dark red) lines represent the 95% confidence intervals for the original estimates for non-Maggi and all noodle purchases respectively (i.e. column 5 in table 2), which are based on the parallel trend assumption. Additional thin lines (blue and red) are the fixed length confidence intervals when allowing for violation of parallel trends of up to 𝑀, which is the maximum possible change in the slope of an underlying purchase trend between each month. Hence, $M=0$ allows for linear deviation.

Figure B3 Parallel trend test of noodle purchases for **infrequent** **buyer**

Note: Each plot presents the estimated average monthly consumer response to the Scandal based on alternative assumptions about the evolution of noodle purchases among infrequent buyer versus comparison group in the absence of the Scandal. The first (dark blue) and the fifth (dark red) lines represent the 95% confidence intervals for the original estimates for non-Maggi and all noodle purchases respectively (i.e. column 6 in table 2), which are based on the parallel trend assumption. Additional thin lines (blue and red) are the fixed length confidence intervals when allowing for violation of parallel trends of up to 𝑀, which is the maximum possible change o in the slope of an underlying purchase trend between each month. Hence, $M=0$ allows for linear deviation.

1. **Placebo tests**

Figure C1 Placebo test – Event study estimates on cracker purchases (in grams) across buyer groups.

| **Monthly Buyer** | **Frequent Buyer** | **Infrequent Buyer** |
| --- | --- | --- |
| 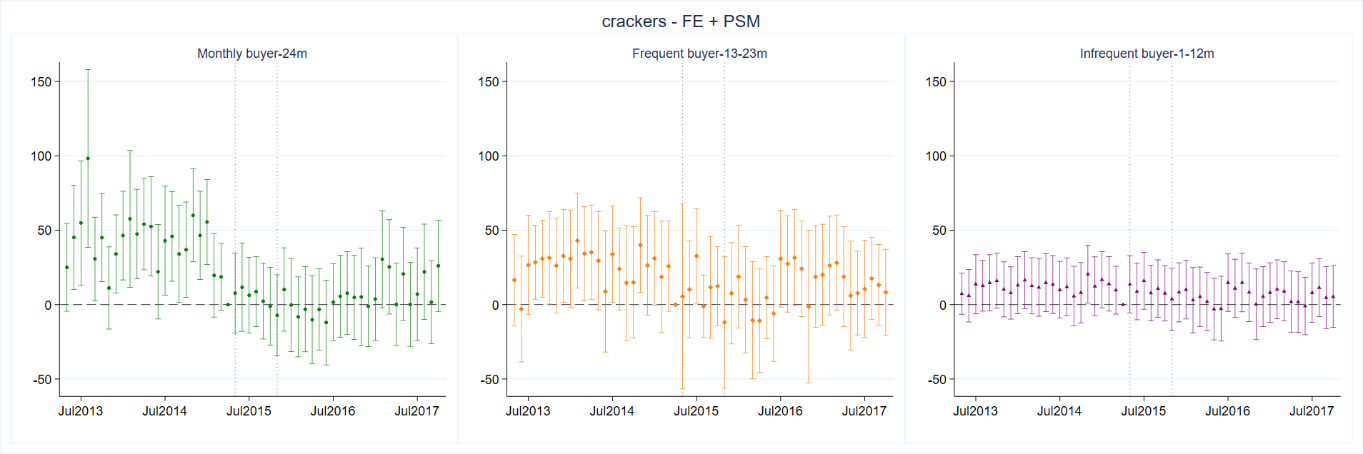 | | |

Note: This figure shows the event study estimates and the 95% confidence interval from equation 1 with inverse propensity weighting on cracker purchases. Standarad errors clustered at household level. Buyer groups are defined based on the number of months they purchased Maggi noodles during the 24 months prior to May 2015: 24 months for Monthly buyer; 13-23 months for Frequent buyer; 1-12 months for Infrequent buyer. Comparsion households are those who did not buy any Maggi noodles 2 years prior to May 2015. Dotted lines indicate the scandal period from May 2015 to November 2015.

Figure C2 Placebo test – Event study estimates on salty snack purchases (in grams) across buyer groups.

| **Monthly Buyer** | **Frequent Buyer** | **Infrequent Buyer** |
| --- | --- | --- |
| 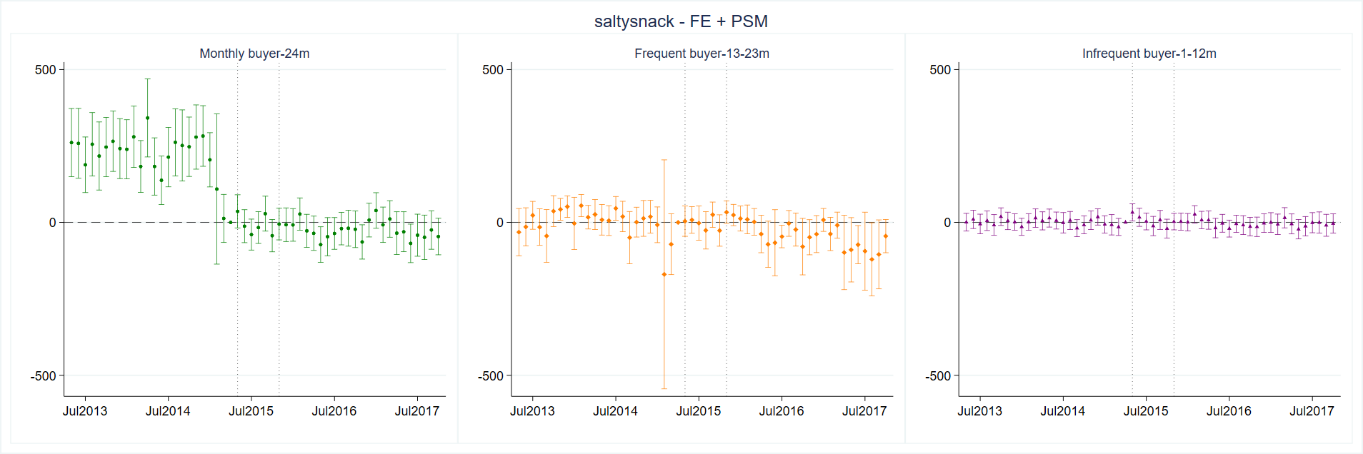 | | |

Note: This figure shows the event study estimates and the 95% confidence interval from equation 1 with inverse propensity weighting on salty snack purchases. Standarad errors clustered at household level. Buyer groups are defined based on the number of months they purchased Maggi noodles during the 24 months prior to May 2015: 24 months for Monthly buyer; 13-23 months for Frequent buyer; 1-12 months for Infrequent buyer. Comparsion households are those who did not buy any Maggi noodles 2 years prior to the Scandal. Dotted lines indicate the scandal period from May 2015 to November 2015.

1. **Synthetic control analysis of wholesale price index (WPI) of noodles in India**

*Specification*

Using the synthetic control approach, we aim to construct a synthetic food item that tracks the WPI of noodles prior to the Scandal. To begin, let $i=1,\ldots,J$ denote the food items covered in the WPI dataset, where $i=1$ corresponds to noodles and $i=2,\ldots,J$ corresponds to each of the $J$ donor food items. We also define $T_{0}$ as the number of months prior to the start of the Scandal with $1\leq T_{0}\leq T$ and $Y_{it}^{N}$ as the WPI outcome that would be observed for any food item $i$ at time $t(t=1,\ldots,T)$ in the absence of the Scandal. $Y_{it}^{E}$ denotes the WPI of food item $i$ after being exposed to the Scandal in the periods $T_{0}$ to $T$. This setup implies that the Scandal has no effect on WPI before $T_{0}$. The approximated change in WPI of noodles due to the Scandal can be estimated through $TE=Y_{it}^{E}-Y_{it}^{N}$.

Following Abadie et al (2010), $Y_{it}^{N}$ can be expressed as a linear function $Y_{it}^{N}= \alpha_{t}+\beta_{t}X_{i}+\gamma_{t}Z_{i}+\varepsilon_{it}$, where $\alpha_{t}$ is a time-dependent factor, $X_{i}$ is a vector of relevant observed pre-treatment covariates, $Z_{i}$ is a vector of unknown covariates, $\beta_{t}$ and $\gamma_{t}$ are unknown vectors of parameters, and $\varepsilon_{it}$ captures random shocks with mean zero.

Now, consider a vector of weights $W=(w_{2},\ldots, w_{J})$ with $w_{i}\geq0$ for $i>1$ and $w_{2}+\ldots+w_{J}=1$. The optimal weight vector, $W^{*}=(w_{2}^{*},\ldots,w_{J}^{*})$, is selected to minimise the mean squared prediction error (MSPE) of the outcome variable in the pre-Scandal period, such that

| $\sum_{i=s}^{J} {w_{i}^{*}Y}_{i1}=Y_{11},\ldots, \sum_{i=s}^{J} {w_{i}^{*}Y}_{iT_{0}}=Y_{1T_{0}} and \sum_{i=s}^{J} w_{i}^{*}X_{i}=X_{1}$ |  |
| --- | --- |

Then, in the absence of a large $\varepsilon_{it}$ and if the above two equations hold,

$$TE=Y_{1t}^{E}-\sum_{i=2}^{J} {w_{i}^{*}Y}_{it}, t>T_{0}$$

To estimate the vector of weights, we use pre-Scandal outcomes of each food item $i$ in the donor pool. Specifically, we construct 3-month averages of the WPI from February 2012 to April 2015 as predictor variables. Table D1 lists all food items in the donor pool and the associated weights chosen based on lowest MSPE. Table D2 shows that there is a strong balance on the lagged WPI predictors between noodles and its synthetic control.

Table D1 Food items and their corresponding weights

| **Food items** | **Weight** | **Food items** | **Weight** |
| --- | --- | --- | --- |
| Almonds | 0.004 | Jowar | 0.002 |
| Amla | 0.004 | Lemon | 0.043 |
| Apple | 0.001 | Maida | 0.002 |
| Arhar | 0.03 | Maize | 0.002 |
| Bagasse | 0.002 | Masur | 0.003 |
| Bajra | 0.001 | Meat of goat | 0.002 |
| Banana | 0.002 | Milk | 0.002 |
| Barley | 0.002 | Molasses | 0.001 |
| Basmati rice | 0.001 | Moong | 0.002 |
| Beans | 0.001 | Mosambi (Sweet Orange) | 0.002 |
| Beef Meat | 0.002 | Mustard Oil | 0.002 |
| Betel Leaves | 0.173 | Mustard oil cake | 0.002 |
| Betelnut/Arecanut | 0.018 | Mutton | 0.003 |
| Biscuit, cookies | 0.002 | Okra (Lady finger) | 0.007 |
| Bitter gourd | 0.003 | Onion | 0.004 |
| Black Pepper | 0.001 | Other meats | 0.002 |
| Bottle gourd | 0.001 | Paddy | 0.002 |
| Bread, buns & croissant | 0.002 | Palm Oil | 0.002 |
| Brinjal | 0.003 | Papaya | 0.057 |
| Buffalo meat | 0.002 | Pear | 0.001 |
| Butter | 0.002 | Peas/Chawali | 0.002 |
| Cabbage | 0.002 | Pineapple | 0.002 |
| Cakes, pastries & muffins | 0.002 | Pointed gourd | 0 |
| Cardamom | 0.004 | Pomengranate | 0.003 |
| Carrot | 0.001 | Pork | 0.001 |
| Cashew nut | 0.004 | Potato | 0 |
| Castor Oil | 0.001 | Poultry Chicken | 0.001 |
| Chicken/duck, dressed | 0.002 | Powder Milk | 0.002 |
| Chillies (Dry) | 0.003 | Processed Tea | 0.002 |
| Chocolate & cocoa powder | 0.003 | Pumpkin | 0.004 |
| Coconut (Fresh) | 0.002 | Radish | 0.016 |
| Coffee | 0.002 | Ragi | 0.002 |
| Coffee powder with chicory | 0.002 | Rajma | 0.001 |
| Condensed Milk | 0.002 | Rapeseed Oil | 0.002 |
| Copra oil | 0.001 | Rice Bran Extract | 0.001 |
| Coriander | 0.001 | Rice Bran Oil | 0.002 |
| Corn Flake | 0.002 | Rice products | 0.002 |
| Cotton seed Oil | 0.002 | Rice, Non-basmati | 0.002 |
| Cotton seed oil cake | 0.002 | Salt | 0.002 |
| Cucumber | 0.005 | Sapota | 0.001 |
| Cumin | 0.011 | Sauces of Vegetables | 0.002 |
| Drumstick | 0 | Shrimps/Prawns | 0.001 |
| Egg | 0.001 | Sooji (rawa ) | 0.002 |
| Fish frozen/canned/processed | 0.004 | Soya preparations excl. oil | 0.001 |
| Fish-Inland | 0.002 | Soyabean Oil | 0.002 |
| Fish-Marine | 0.002 | Spices | 0.003 |
| Flour of other cereals | 0.002 | Sugar | 0.001 |
| Fruit Juice | 0.002 | Sugar confectionary | 0.002 |
| Fruit pulp | 0.003 | Sugarcane | 0.002 |
| Garlic | 0.056 | Sunflower Oil | 0.003 |
| Ghee | 0.002 | Sweet Potato | 0.001 |
| Ginger (Dry) | 0.001 | Tamarind | 0.01 |
| Ginger (Fresh) | 0.001 | Tapioca | 0.001 |
| Gola & similar Cattle Feed | 0.002 | Tea | 0.001 |
| Gram | 0.004 | Tomato | 0.007 |
| Gram powder (besan) | 0.026 | Turmeric | 0.151 |
| Groundnut Oil | 0.004 | Urad | 0.004 |
| Guava | 0.001 | Vanaspati | 0.002 |
| Gur | 0.002 | Vegetable starch | 0.002 |
| Honey | 0.002 | Walnut | 0.001 |
| Ice cream | 0.002 | Wheat | 0.002 |
| Instant Coffee | 0.002 | Wheat Bran | 0.002 |
| Vegetable-based Instant Food/Prepared meals | 0.002 | Wheat flour (Atta) | 0.002 |
| Jackfruit | 0.166 | Whey powder | 0.002 |
| Jams, jellies, puree | 0.002 |  |  |

Table D2 Predictor balance for Noodles and its synthetic control

| Predictors | Treated | Synthetic |
| --- | --- | --- |
| 3-month average WPI during the following period |  |  |
| February 2013 – April 2013 | 110.37 | 110.41 |
| May 2013 – July 2013 | 109.60 | 109.69 |
| August 2013 – October 2013 | 111.00 | 111.10 |
| November 2013 – January 2014 | 114.70 | 114.70 |
| February 2014 – April 2014 | 117.07 | 117.13 |
| May 2014 – July 2014 | 110.93 | 110.95 |
| August 2014 – October 2014 | 114.97 | 114.93 |
| November 2014 – January 2015 | 120.03 | 119.93 |
| February 2015 – April 2015 | 128.70 | 128.70 |

*Placebo test*

Abadie et al. (2010) recommend using placebo tests, which are similar to permutation tests, to assess the “statistical significance” of the estimated effect. This is implemented by iteratively running the synthetic control procedure on each food item of the donor pool as if it were the food item affected by the Scandal while the remaining items serve as the donor pool. The thin grey lines in figure D3 shows the distribution of the difference in WPI of each food item in the donor pool and its respective synthetic version. Since the estimated gap for WPI of noodles and its synthetic control (thick blue line) lies in the middle of the distribution, there is more confidence that noodle prices did not change significantly by the Scandal.

Figure D3 Difference in WPI of food items and the respective synthetic WPI

Note: The thick blue line shows the estimated gap between the WPI of noodles and its synthetic control. The grey lines give the estimated gap for each of the other food items in the donor pool.

1. **Heterogeneous changes in noodle purchases in response to the Scandal (in grams/month)**

| **Buyer group** | **Monthly buyer** | |  | **Frequent buyer** | |  | **Infrequent buyer** | |
| --- | --- | --- | --- | --- | --- | --- | --- | --- |
| Total purchase of Maggi noodles during the 24 months prior to 2015 (The threshold corresponds to the average total purchase within each buyer group) | Above 12.83kg | Below 12.83kg |  | Above 7.26kg | Below 7.26kg |  | Above 1.68kg | Below 1.68kg |
|  | (1) | (2) |  | (3) | (4) |  | (5) | (6) |
| *Panel A: Non Maggi Noodles (Reference period: April 2015)* | | | | | | | | |
| Immediate response | 81.48*** | 51.22*** |  | 31.45*** | 10.81*** |  | -13.53*** | -4.62 |
| (May 2015 - Nov 2015) | (10.10) | (5.23) |  | (4.67) | (3.47) |  | (3.85) | (3.29) |
| 1-year post response | 87.86*** | 64.25*** |  | 46.58*** | 30.34*** |  | 6.84* | 0.48 |
| (Dec 2015 - Nov 2016) | (10.19) | (5.72) |  | (4.61) | (3.39) |  | (3.82) | (3.19) |
| 2-year post response | 30.91*** | 37.57*** |  | 9.088* | 17.91*** |  | -7.39* | -3.21 |
| (Dec 2016 -Nov 2017) | (9.72) | (5.44) |  | (4.71) | (3.62) |  | (4.04) | (3.41) |
| *Panel B: All noodles (Reference period: April 2015)* | | | | | | | | |
| Immediate response | -549.15*** | -241.36*** |  | -329.76*** | -140.36*** |  | -122.05*** | -34.26*** |
| (May 2015 - Nov 2015) | (26.69) | (10.12) |  | (6.82) | (3.86) |  | (4.45) | (3.35) |
| 1-year post response | -236.65*** | -97.46*** |  | -171.90*** | -73.41*** |  | -71.60*** | -24.01*** |
| (Dec 2015 - Nov 2016) | (26.17) | (10.55) |  | (6.74) | (3.81) |  | (4.47) | (3.31) |
| 2-year post response | -222.65*** | -59.05*** |  | -142.63*** | -48.52*** |  | -58.75*** | -22.83*** |
| (Dec 2016 -Nov 2017) | (28.48) | (10.82) |  | (7.11) | (4.21) |  | (4.82) | (3.68) |
|  |  |  |  |  |  |  |  |  |
| Household fixed effect | Yes | Yes |  | Yes | Yes |  | Yes | Yes |
| N | 169,452 | 154,062 |  | 745,848 | 524,610 |  | 923,400 | 630,072 |

Note: This table presents unweighted average event study estimates of the corresponding period. Buyer groups are defined based on the number of months they purchased Maggi noodles during the 24 months prior to May 2015: 24 months for Monthly buyer; 13-23 months for Frequent buyer; 1-12 months for Infrequent buyer. Within each buyer group, we compute the average total purchase of Maggi noodles during the 24 months prior to May 2015, which is then used to further classify households into above and below average purchase of Maggi noodles. The threshold is computed seperately for each buyer group. Comparsion households are those who did not buy any Maggi noodles for 2 years prior to May 2015. Robust standard errors clustered at household level. ***p<0.01 **p<0.05 *p<0.1

1. The abbreviation SD stands for second derivatives. [↑](#footnote-ref-2)
2. For example, Rose (2021) and Ang (2021) use this approach to illustrate the “breakdown” point of their results, which is the magnitude of non-linear post-treatment violations of parallel trends (i.e. M) needed for their estimates to become statistically insignificant. Dustmann et al (2021) show that their findings of no minimum-wage induced disemployment effects as well as the positive wage effects continue to hold after allowing 20% per year non-linear deviations from the pre-trend in the two post-policy years. [↑](#footnote-ref-3)
